# Discovery of the Involvement in DNA Oxidative Damage of Human Sperm Nuclear Basic Proteins of Healthy Young Men Living in Polluted Areas

**DOI:** 10.3390/ijms21124198

**Published:** 2020-06-12

**Authors:** Gennaro Lettieri, Giovanni D’Agostino, Elena Mele, Carolina Cardito, Rosa Esposito, Annalinda Cimmino, Antonella Giarra, Marco Trifuoggi, Salvatore Raimondo, Tiziana Notari, Ferdinando Febbraio, Luigi Montano, Marina Piscopo

**Affiliations:** 1 Department of Biology, University of Naples Federico II, 80126 Napoli, Italy; gennarole@outlook.com (G.L.); giov.dago@gmail.com (G.D.); elena.mele94@gmail.com (E.M.); carolina.cardito17@gmail.com (C.C.); rosa-expo@hotmail.it (R.E.); 2 CNR, Institute of Biochemistry and Cell Biology, via Pietro Castellino, 80131 Naples, Italy; cimmino.annalinda88@gmail.com; 3 Department of Chemical Sciences, University of Naples Federico II, Via Cinthia, 21, 80126 Naples, Italy; antonella.giarra@unina.it (A.G.); marco.trifuoggi@unina.it (M.T.); 4 Seminology Unit Gentile Research Centre, 80054 Gragnano, Italy; salvatoreraimondo57@gmail.com; 5 GEA—Gynecology Embryology Andrology—Reproductive Medicine Unit of Check Up Polydiagnostic Center, 84131 Salerno, Italy; tiziananotari7@gmail.com; 6 Andrology Unit of the “S. Francesco d’Assisi” Hospital, Local Health Authority (ASL) Salerno, EcoFoodFertility Project Coordination Unit, 84020 Oliveto Citra, Italy

**Keywords:** DNA oxidative damage, protein–DNA binding, human protamines, heavy metals, EMSA, fluorescence

## Abstract

DNA oxidative damage is one of the main concerns being implicated in severe cell alterations, promoting different types of human disorders and diseases. For their characteristics, male gametes are the most sensitive cells to the accumulation of damaged DNA. We have recently reported the relevance of arginine residues in the Cu(II)-induced DNA breakage of sperm H1 histones. In this work, we have extended our previous findings investigating the involvement of human sperm nuclear basic proteins on DNA oxidative damage in healthy males presenting copper and chromium excess in their semen. We found in 84% of those males an altered protamines/histones ratio and a different DNA binding mode even for those presenting a canonical protamines/histones ratio. Furthermore, all the sperm nuclear basic proteins from these samples that resulted were involved in DNA oxidative damage, supporting the idea that these proteins could promote the Fenton reaction in DNA proximity by increasing the availability of these metals near the binding surface of DNA. In conclusion, our study reveals a new and unexpected behavior of human sperm nuclear basic proteins in oxidative DNA damage, providing new insights for understanding the mechanisms related to processes in which oxidative DNA damage is implicated.

## 1. Introduction

DNA is the vital carrier of genetic information in all living cells, but its chemical stability is affected by several factors. In fact, DNA is highly susceptible to chemical modifications by exogenous agents such as ionizing radiation and ultraviolet light [1,2,3] and by several environmental contaminants (pesticides, hydrocarbons, and especially heavy metals), which can generate oxidative stress [4,5]. Beyond environmental agents, DNA is also subject to oxidative damage from by-products of cellular metabolism (endogenous agents). The consequential alterations of DNA structure are generally incompatible with its crucial role in the maintenance and transmission of genetic information. For this reason, cells respond to DNA oxidative damage by specified DNA repair pathways to physically remove the damages [6]. In the past 20 years, many papers regarding the involvement of DNA oxidative damage in human infertility have been published [7]. It is well known that both male and female gametes can be exposed to DNA damage, which may compromise their functionality and their capacity to produce normal embryos. In particular, DNA damage, affecting sperm quality, increases the risk of genetic and epigenetic abnormalities and can lead to some diseases. Although a small amount of Reactive Oxygen Species (ROS) is necessary for some fundamental processes for the physiological function of male gametes, high levels of ROS can cause functional failure [8]. There is a great interest in having new insights in the mechanisms of DNA oxidative damage, since it can also cause genetic alterations that may result in diseases, such as cancer and neurodegenerative syndromes [9], and contribute to some features of aging. Despite the numerous studies conducted on the matter [10], the precise molecular mechanisms that lead to DNA oxidative damage are not yet fully understood. In particular, to the best of our knowledge, no previous study has evaluated the possible involvement of Sperm Nuclear Basic Proteins (SNBP) in DNA oxidative damage. As a rule, histones and protamines, compacting DNA, protect from oxidative damage. Thus, in some stress conditions, possible functional alterations of SNBP properties should be more evident, considering the higher degree of compaction of sperm chromatin compared to that of somatic cells. As a matter of fact, in a previous work, we have demonstrated that some sperm histones, in the presence of specific heavy metals, can participate in DNA oxidative damage [4], suggesting that in particular stress conditions, their protective rule appears to be reversed. This observation prompted us to investigate through molecular-level analyses the possibility that SNBP from men living in polluted areas might have an involvement in oxidative DNA damage. In fact, we found a higher DNA fragmentation index in the spermatozoa of males recruited in the “Land of Fires”, which is a high environmental impact area of Campania Region (Southern Italy) [11,12,13,14] in which resident people presented similar values for semen volume, pH, sperm cell count, and morphology, but a significant increase of immotile cells percentage [11].

To this aim, we have evaluated the protein framework, the DNA binding, and the potentiality to induce the oxidative DNA damage of SNBP from a cohort of men living in the “Land of Fires”. This study was conducted as part of a biomonitoring project “EcoFoodFertility” [11] (http://www.ecofoodfertility.it/the-project.html).

## 2. Results

### 2.1. Characteristics of Impacted Areas Used for the Recruitment

**Figure 1:**
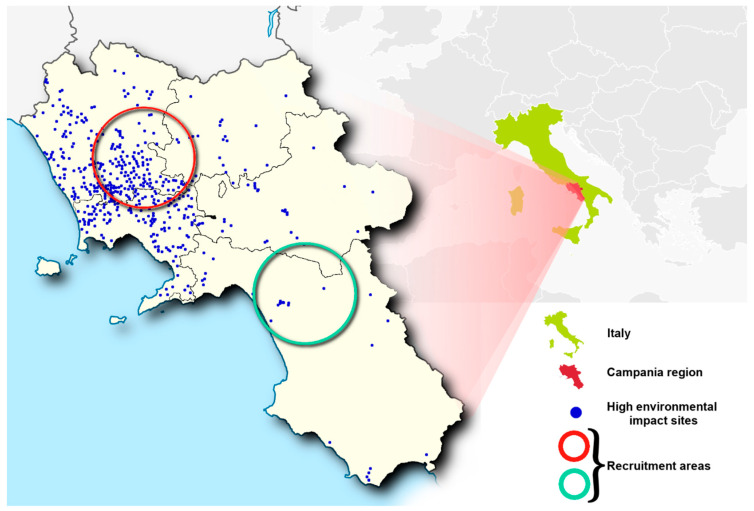
Map of the geographical areas selected for the recruitment. The red and green circles indicate the high and low environmental impact areas, respectively, in Campania region (Italy). Blue points indicate the sites of high environmental impact as recognized by the Campania Region Environmental Protection Agency report (2008).

The geographical areas selected for the recruitment (Figure 1) differ for the number of sites recognized by the Campania Region Environmental Protection Agency for the presence of a high concentration of toxic contaminants [15]. We considered 9 municipalities, inside the red circle in Figure 1, showing a high number of illegal disposal sites of toxic waste (Table 1). It’s interesting to note that in the pattern of chemical elements of semen from males living in this area, the presence of high concentrations of Cr, Cu, and Zn was found [11]. The 7 municipalities in the area included in the green circle in Figure 1 were considered for the low number of environmental impact sites.

### 2.2. Analysis of SNBP

**Figure 2:**
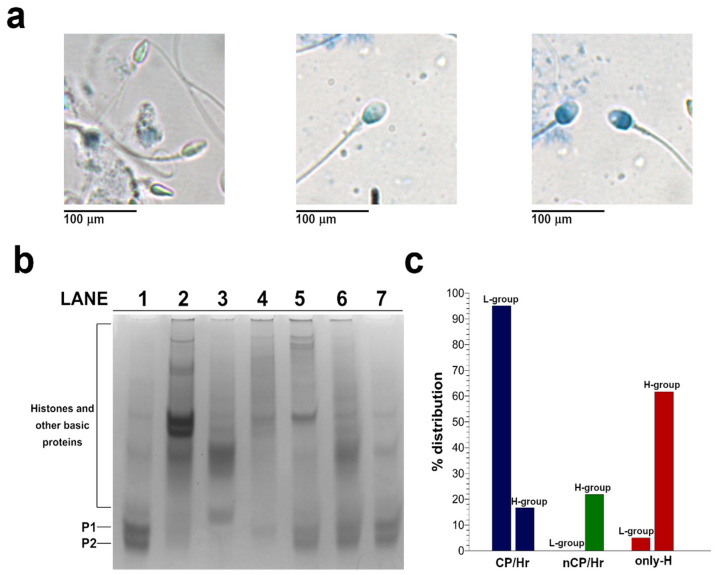
Characterization of human Sperm Nuclear Basic Proteins (SNBP) from samples belonging to the H- and L-groups. (**a**) Staining of spermatozoa collected from samples of H-group males. The light (left), the intermediate (middle), and dark aniline blue-stained (right) spermatozoa represent mature, moderately immature, and severely arrested maturity spermatozoa found in this group. (**b**) AU-PAGE of SNBP showing the CP/Hr (lane 1), only-H (lane 2–3), and not CP/Hr (lanes 4–7). (**c**) Percentage distribution in H-and L-groups of protamines/histones ratios found in spermatozoa. In blue, the samples containing the CP/Hr; in green, the nCP/Hr; and in red, the samples showing only-H.

We have observed different staining in the spermatozoa collected from samples of males living in the high (H-group) environmental impact area (Figure 2a). Regarding the nuclear maturity, the light, the intermediate, and the dark aniline blue-stained spermatozoa represent mature, moderately immature, and severely arrested maturity spermatozoa, as previously described [16]. We found a prevalence of samples containing dark blue-stained spermatozoa (right panel in Figure 2a), indicating the persistence of histones in the chromatin. In lower number of samples, we detect a prevalence of light and intermediate blue-stained spermatozoa (left and middle panels in Figure 2a, respectively), indicating mature and immature gametes, respectively. Instead, almost all the males living in the low (L-group) environmental impact areas had light-stained spermatozoa in their samples, showing the prevalence of mature gametes. We extracted the SNBP from samples belonging to males living in the two areas and characterized the protein content by Acid-urea Polyacrylamide Gel Electrophoresis (AU-PAGE) (Figure 2b). In lane 1 of Figure 2b, a representative electrophoretic pattern of samples belonging to the L-group is shown. In this sample, we observed the classic electrophoretic pattern of human SNBP, with the canonical protamines/histones ratio (CP/Hr), which was accordingly previously described [17]. In the samples of males belonging to the H-group, we observed several differences in the electrophoretic protein patterns. In particular, we identified samples showing only histones and other basic proteins (only-H) (lanes 2 and 3 in Figure 2b) in which protamines seem to be absent. In another group of samples, we observed the presence of protamines and histones, but not in the canonical ratio (nCP/Hr). This latter condition was very heterogeneous, presenting a variable ratio between protamines and histones, as shown in Figure 2b (lanes 4–7). We categorized the analyzed 240 samples belonging to the two different areas based on the number and type of bands identified on the AU-PAGE, which is in accordance with the classification reported in [17]. These three types of electrophoretic profiles were differently distributed in the L- and H-groups. In particular, in males belonging to the L-group, we found only two conditions, the first represented by the 95.06% of samples shoving the CP/Hr profile, and the other represented by the 4.94% of samples showing the only-H pattern (Figure 2c). Differently, we found the CP/Hr profile in the 16.61% of sample from males belonging to the H-group, observing in the majority of this group samples (61.61%) the only-H profile. The remaining 21.78% of samples from the H-group showed a heterogeneous nCP/Hr profile (Figure 2c). The presence of bands close to the well in the samples showing altered protamine/histone ratios could be caused by the presence of substances promoting protein aggregation, such as the excess of some chemicals found in the semen of people from Land of Fire [11].

### 2.3. DNA Binding Ability of SNBP Analyzed by EMSA

We studied, by Electrophoretic Mobility Shift Assays (EMSA), the differences in the ability to bind the DNA of the three typologies of SNBP observed in the samples of the L- and H-groups. In particular, we evaluated the ratio proteins/DNA necessary to obtain DNA saturation, which was indicated by the formation of a high-molecular weight DNA band, close to the well, in electrophoretic pattern [18]. All the analyzed protein samples interacted with DNA in the typical “all or nothing” DNA binding mode of SNBP in agreement with data previously reported for SNBP [19,20]. Briefly, in the “all or nothing” DNA binding mode, the DNA band on the gel migrates with high mobility in the absence or in the presence of low protein (histones or protamines) concentrations. At increasing protein/DNA ratios, there are no DNA bands with intermediate electrophoretic mobility, but there begins to appear a DNA band with low mobility, close to the well, which is indicative of high molecular weight complex DNA–proteins.

**Figure 3:**
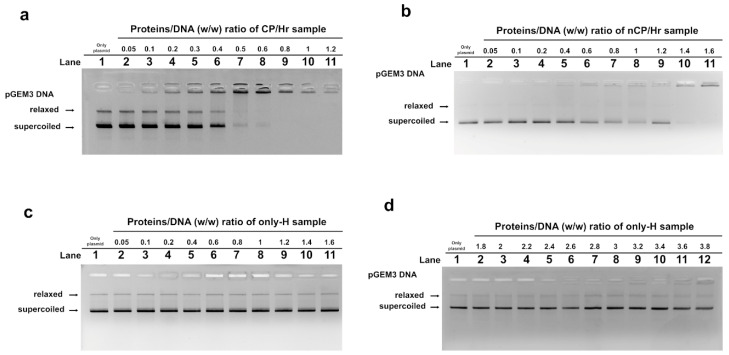
DNA-binding ability of SNBP obtained from H-group analyzed by Electrophoretic Mobility Shift Assays (EMSA) on 1% agarose gel. Bands on gel representing the state of pGEM3 plasmid DNA incubated in a ratio *w*/*w* with increasing amount of SNBP from samples containing CP/Hr (**a**), nCP/Hr (**b**), and only-H (**c**,**d**).

We found that protein samples presenting the CP/Hr profile belonging to the H-group reached DNA saturation at a protein/DNA ratio of about 0.8 (Figure 3a), instead of the 1.2 ratio observed for the SNBP from samples of the L-group (Figure S1). As expected, samples presenting the only-H profile, regardless of whether they belonged to the L- and H-groups, showed instead low DNA binding ability, because DNA saturation did not occur even at 3.8 proteins/DNA ratio (Figure 3c,d). In accordance with the heterogeneity of the samples belonging to the third condition (nCP/Hr), we observed similar trends, but not identical DNA saturation values. However, all samples presented common features, showing a reduced DNA binding ability and a not-stable binding mode to DNA. In fact, as shown in the representative gel of Figure 3b, we observed, at the protein/DNA ratio concentrations from 0.05 to 1 (lanes 2–8), the decrease of supercoiled plasmid DNA fraction and the increase of the fraction close to the well. The next addition of proteins at protein/DNA ratio 1.2 (lane 9) results in an increase of supercoiled DNA fraction suggesting proteins detachment to DNA (compare lanes 8 and 9 in Figure 3b).

### 2.4. DNA Binding Ability of Sperm Proteins Determined by Fluorescence Analysis

**Figure 4:**
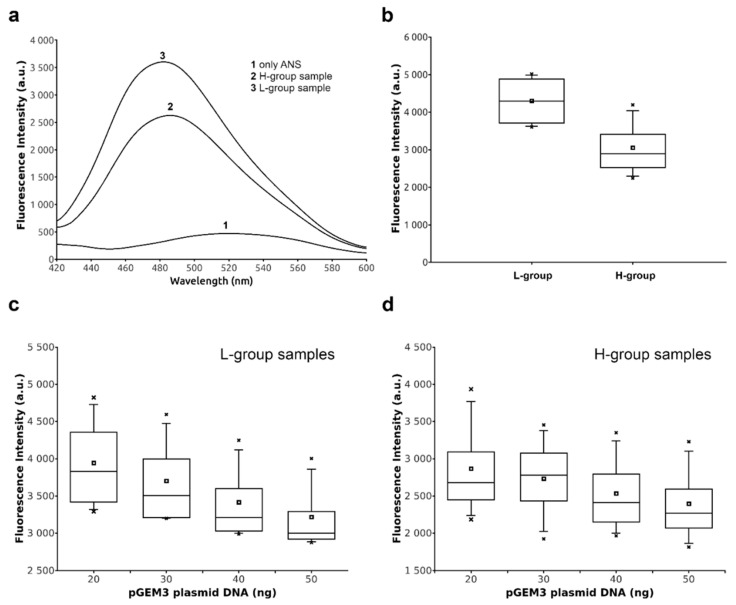
Fluorescence analyses of SNBP from samples containing CP/Hr belonging to the H- and L-groups. (**a**) Fluorescence spectra of ANS in the absence (curve 1) and in the presence of SNBP obtained from samples belonging to H- (curve 2) and L-groups (curve 3). (**b**) Box plot of fluorescence intensities of 8-anilinonaphthalene-1-sulfonic acid (ANS) in the presence of SNBP from samples belonging to H- and L-groups. Box plot of fluorescence intensities of the complexes ANS–SNBP belonging to the L-group (**c**) and H-group (**d**), in the presence of increasing pGEM3 plasmid DNA concentrations.

Considering that the anomalous behaviors of the only-H and nCP/Hr conditions can easily explain the differences observed in their binding to DNA, we focused on the slight differences observed in the CP/Hr samples belonging the L- and H-groups. We analyzed the features of these latter in a more detailed manner by using extrinsic fluorescence approaches in solution. We exploited the sensitivity of fluorescent probe 8-anilinonaphthalene-1-sulfonic acid (ANS) to solvent polarity, increasing its fluorescence intensity on binding to biomolecules, in addition to a strong solvent-dependent shift in its emission spectrum [21,22]. We observed an increase in the fluorescence of ANS in the presence of sample belonging to the L-group with respect the ones from H-group; in addition, a marked blue-shift of the maximum of fluorescence was recorded for the sample collected in the low-environmental impact areas (Figure 4a). This trend was observed for all the fluorescence-measured samples belonging the two groups, as shown by the box plot in Figure 4b. The observed difference was sufficiently significant, with a measured *p*-value ≤ 0.05, indicating a different accessibility of the fluorescent probe to the surface of proteins belonging to the two groups. These differences also affected the ability to bind the DNA of the proteins isolated from the two groups. In particular, measurements of ANS fluorescence in the presence of the complex protein–DNA of the proteins belonging to the L-group showed a linear decrease of the fluorescence at the increase of DNA concentration (Figure 4c). This result can be explained by a reduced protein surface available to the binding with the fluorescent probe, being the protein constantly subtracted by the binding to DNA. Although, also in the sample belonging to the H-group, we observed a similar decrease in the ANS fluorescence after the addition of increasing concentrations of DNA, the slope was not linear, but better described by a second-order function (Figure 4d). The differences in the overall fluorescence and in the interaction mode of human protamines belonging to the H-group suggested changes in the exposed surface of these proteins.

### 2.5. H_2_O_2_-Induced DNA Breakage in the Presence of Human SNBP

**Figure 5:**
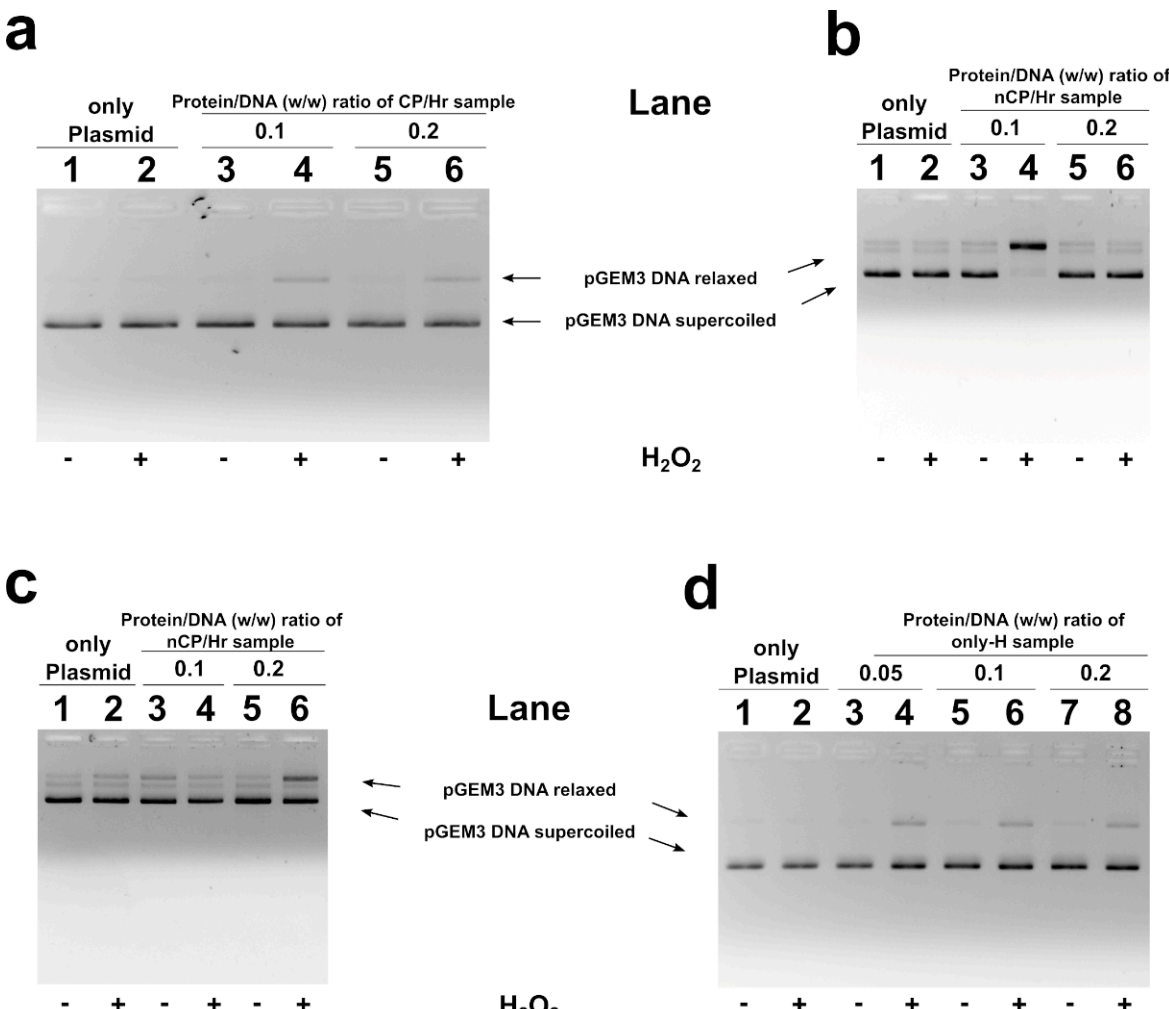
Analysis on 1% agarose gel of pGEM3 plamid DNA breakage induced by H_2_O_2_. In the presence of SNBP from H-group samples showing CP/Hr (**a**), nCP/Hr (**b**,**c**) and only-H (**d**).

In Figure 5, the results of the analyses of H_2_O_2_-induced DNA breakage in the presence of human SNBP are shown. DNA breakage was evaluated by the conversion of a supercoiled to relaxed form of pGEM3 DNA plasmid in the presence of SNBP. In our experimental conditions, DNA breakage was not observed when plasmid was mixed with 30 μM H_2_O_2_, being necessary higher H_2_O_2_ concentration, at least 100 µM, in order to cause DNA breakage (Figure S2). The addition of samples containing CP/Hr, at low protein/DNA ratios, to the pGEM3 DNA plasmid in the presence of H_2_O_2_ resulted in an increase of the relaxed plasmid DNA fraction at detriment of the supercoiled one (Figure 5a lanes 4, 6). Similar results were observed also for samples belonging to the H-group containing only-H (Figure 5d lanes 4, 6, 8). More relevant DNA damage was observed by using samples containing nCP/Hr; in fact, in this latter case, plasmid DNA appeared almost completely in the relaxed form (Figure 5b, lane 4). However, this latter condition being very heterogeneous, we have also observed differences in the protein/DNA ratio necessary to obtain DNA damage (Figure 5c, lane 6). The same analysis performed with samples of males living in low environmental areas presenting protamines and histones in canonical ratio did not show DNA breakage (Figure S3).

## 3. Discussion

Living organisms are constantly exposed to numerous DNA damaging agents that can impact health and modulate disease states. DNA damage can cause genetic alterations that lead to the development of cancer, may result in cell death, as in neurodegenerative diseases, and could contribute to some features of aging [2]. However, the highest risk of DNA damage is represented by the effects at the gametes level, since this can jeopardize the possibility of fertilization and consequently the continuity of the species. Indeed, due to their characteristics, male gametes are the most sensitive cells to the accumulation of damaged DNA, considering their continuous production and exposure to environmental agents, such as oxidizing agents [23,24,25,26]. In this regard, many studies have been performed to understand the mechanisms of oxidative DNA damage, but some aspects are still unknown. We have tried to give new insights on this topic using human spermatozoa as model cell for this study, evaluating the possible involvement of human SNBP in DNA oxidative damage. In fact, we have already reported that in the presence of heavy metals, some SNBP isolated from other organisms can participate in DNA oxidative damage, reversing their canonical protective rule [4]. However, in vitro studies are not sufficient to describe the complexity of in vivo effects on DNA oxidative damage, because DNA is not free but complexed with proteins to form chromatin in living cells [27], and it has been observed that Cu (II)/H_2_O_2_-induced DNA damage increases in the nucleosome compared to isolated DNA [28]. Therefore, it is of fundamental importance to conduct in vivo studies on DNA oxidative damage, since the results that can be obtained are an order of magnitude more complex than those obtained by treating only DNA in vitro. These observations are in agreement with our in vivo studies on SNBP from mussels exposed to subtoxic concentrations of heavy metals, such as copper or cadmium [5,29]. In fact, we found that the heavy metals measured in gonads accumulated mainly in the fraction of SNBP, causing their involvement in DNA oxidative damage [5,30]. Therefore, in the present study, we considered it more appropriate to use the spermatozoa of men living in high environmental impact sites, such the “Land of Fires”, where some heavy metals, that participate in Fenton-like reactions, such as copper or chromium, are particularly abundant. In fact, in these subjects, a higher DNA fragmentation index [12] and alterations of specific bio-markers of DNA oxidative damages have been reported [11]. In particular, the comparison of subsets of randomly selected subjects from the L- and H-groups showed significantly lower Glutathione-S reductase and Glutathione peroxidase activities in the subset from the H-group (−32% and −25%, respectively; *p* < 0.05). Moreover, the mRNA level of γ-Glutamate cysteine ligase was also two-fold lower in the latter subset. In addition, DNA damage was measured in the same subsets, where antioxidant enzymes were assessed, reporting a DNA fragmentation index (DFI) value 2-fold higher in the H-group with respect to the L- one (*p* = 0.01) [11].

**Figure 6:**
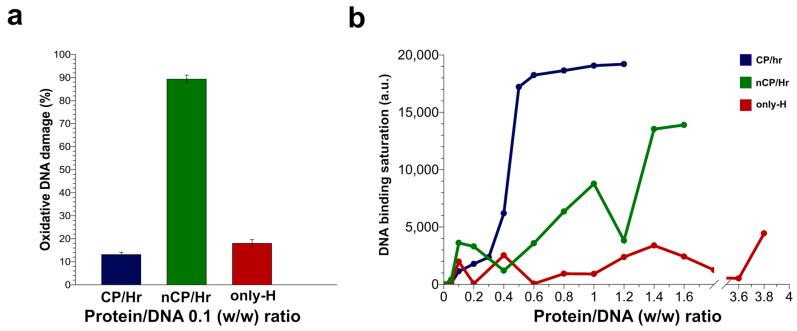
Graphical representation of the effects on DNA of human sperm proteins from samples belonging to the H-group showing canonical protamines/histones ratio (CP/Hr), protamines and histones, but not in the canonical ratio (nCP/Hr), and only-H. (**a**) Histograms of DNA oxidative damage, at 0.1 protein/DNA *w*/*w* ratio, quantified by densitometric analysis of bands on gels in Figure 5. (**b**) Lines chart of the DNA binding saturation, at increasing protein/DNA ratios, calculated by densitometric analysis of supercoiled bands on the gels of Figure 3.

In agreement, our molecular analyses indicated an unusual distribution in the electrophoretic profiles of SNBP in men belonging to the H-group. In addition, we observed that all SNBP isolated from this group changed their protective ability, participating in DNA oxidative damage. This result was particularly marked for those samples presenting a not canonical protamine/histone ratio (Figure 6a). This strong occurrence of DNA oxidative damage in samples from men belonging to the H-group could be explained by an excess of copper and chromium found in the semen of people living in the “Land of Fires” [11]. In fact, it is well known that copper overload generally leads to oxidative stress, promoting the formation of hydroxyl radicals, which strongly reacts with practically any biological molecule, including DNA, causing severe damage to the cells [31,32,33,34]. Several studies have also demonstrated that copper can form several binary and ternary complexes with arginine residues [35,36,37], of which human protamines are extremely rich, promoting a site-specific damage at guanine residues of DNA by a selective binding between guanine and arginine [38]. Moreover, our recent studies have also revealed that Cu(II) interacts with arginine residues of sperm H1 histones, inducing oxidative DNA damage [4]. Moreover, Human Protamine 2 has a strong Cu(II)-binding amino acid motif at its N-terminus (Arg-Thr-His), which is able to mediate oxidative DNA double-strand scission and the generation of 8-oxo-2′-deoxyguanosine (8-oxo-dG) from free 2′-deoxyguanosine (dG) and from DNA by H_2_O_2_ [39,40]. Keeping in mind this evidence, it would be possible to speculate that these proteins could trap this metal, increasing the availability of Cu(II) ions near the binding surface of DNA. This condition could have as a consequence the promotion of the Fenton reaction in DNA proximity after H_2_O_2_ addition, determining DNA breakage and explaining the DNA oxidative damage found in CP/Hr samples of men belonging to the H-group. This finding is in accordance with the analyses carried out in the presence of Cu(II) concentrations for the in vitro determination of DNA-binding affinity of protamines and their involvement in DNA breakage. In fact, preliminary experiments indicated an increase in the DNA-binding affinity of CP/Hr proteins belonging to the L-group, in the presence of copper chloride, saturating the DNA at the protein/DNA ratio of 0.3 (lane 5, Figure S4) instead of 1.2, as observed for the CP/Hr proteins samples of the L-group in the absence of copper chloride (lane 9, Figure S1). In addition, in the presence of copper chloride, we observed an increase of relaxed DNA plasmid (lanes 4 and 10, Figure S5), confirming the involvement of protamines in the DNA oxidative damage, as already demonstrated in our previous work on sperm H1 histones [4]. We found in the literature that also chromium, the other heavy metal found in excess in the semen of people in the H-group, could participate in Fenton-like reactions producing reactive oxygen species and could influence the structure of chromatin by binding to both DNA and histones [41,42]. The toxic effect of chromium results in radical-mediated DNA strand breakage and the formation of stable chromium–DNA complexes, including chromium–DNA adducts and protein–chromium–DNA and DNA–chromium–DNA cross-links [43,44]. In addition, histones bind chromium through lysine residues [41,45] and could determine an “indirect” DNA damage in a similar way as hypothesized for copper. These evidences could explain the effect measured in the samples from men belonging to the H-group, showing the presence of only histones. In fact, in these samples, we observed an extent of damage comparable to that found in the samples belonging to the men of the H-group, showing a canonical protamine/histone ratio (Figure 6a).

The concomitant presence in the area under study of an excess of these heavy metals, participating in the Fenton reaction and able to bind histones and protamines respectively, can justify the more marked extend of DNA oxidative damage measured in the samples presenting histones and protamines in not canonical ratio (Figure 6a). The ability to induce DNA breakage, observed in the SNBP of men belonging to the H-group, can also be ascribed to the structural changes of these proteins due to tertiary/quaternary structure interactions. In order to study these conformational changes, we performed fluorescence measurements that are a sensitive tool to obtain information about protein–ligand interactions. However, we could not use the intrinsic fluorescence of these proteins because of their low content in aromatic amino acids. Therefore, we analyzed the features of sperm nuclear basic proteins in the samples showing the canonical ratio protamines/histones in the L and H-groups by using extrinsic fluorescence approaches with a solvatochromic dye such as ANS [21]. It is known that solvatochromic dyes are powerful tools for monitoring protein conformational changes and proteins interactions with nucleic acids, other proteins, and lipid membranes [22]. Generally, the increase of ANS fluorescence intensity and a blue shift in the emission maxima are attributed to the binding of the fluorescent probe to the hydrophobic sites on the protein and to its reduced mobility [21]. However, it has been also reported that ANS could bind arginine and lysine residues on the protein surface through ion pair formation [46], although the total fluorescence contribution of the ANS bond to these external sites is much less compared to that from the buried sites. In our experiments, we observed a reduced fluorescence intensity of ANS in the sample from the H-group with respect to the ones from the L-group, indicating a different accessibility of the fluorescent probe to the surface of proteins belonging to the two groups. Taking into account the high extend of basic amino acid residues (arginine, lysine, and histidine residues) on the surface of these proteins, we could explain this outcome by a lesser number of arginine residues that could bind ANS in the sample from the H-group, following the binding with heavy metals. Otherwise, we can also hypothesize an indirect effect on the ANS fluorescence of the binding of protamines with the heavy metals. In fact, the increment of the total surface charge of the H-group protamines due to the addition of the positive charges of heavy metals could result in a more hydrophilic dielectric constant of the solution, quenching the fluorescence of the solvatochromic dye. Either of these hypotheses supported possible changes in the function of protamines, being the protein surface altered. In fact, these differences also affected the ability to bind the DNA of sperm protein from samples belonging to the H-group, having measured a not linear fluorescence quenching at the increased DNA concentrations with respect to the ones of the L-group. These differences in DNA binding were more evident from the plot of the band density against the protein/DNA ratio (Figure 6b). In fact, analyzing the DNA-binding ability of the SNBP from the samples of the H-group, we observed that DNA saturation was reached using a lower amount of proteins with respect to the SNBP from the samples of the L-group. This behavior supports the hypothesis of a possible alteration in the proteins’ surface with an overall increase in the positive charge of the protein mediated by surface ions, determining a strong bond to DNA.

These differences in DNA binding could also explain the behavior of the samples with nCP/Hr (Figure 6b). The high content in arginine residues of protamines permits the binding both to minor and major DNA grooves, producing the adequate degree of sperm chromatin compactness, while histones interact only with the precise region of sperm DNA, producing a less compact chromatin [47]. Taking into account that 10–15% of histones are retained in human sperm chromatin [48,49,50,51], forming a heterogeneous mixture of nucleohistones and nucleoprotamines, we could hypothesize that the presence of both protamines and histones in altered ratios could determine not only an unstable binding to DNA, but also a reduced DNA protection to the external agents, such as heavy metals. In addition, in samples showing only histones, the low degree of compactness of the sperm chromatin could result in a low amount of chromium presented locally to DNA by histones, but at the same time, an increased exposure of DNA to external perturbants. Accordingly, increased concentrations of only histones samples resulted in a decreased degree of DNA breakage (Figure S6). As regards chromium, it is also important to consider that growing evidence suggests that epigenetic effects may in part be dependable for their genotoxicity and carcinogenicity [52,53]. In fact, it has been demonstrated that long-term chromium exposures may cause a significant increase in histone deacetylation. This effect may be particularly relevant in the histones–protamines transition which, as well known, requires histone acetylase activity and then could explain the high percentage of subjects (about 65%) in H areas that presented only histones in spermatozoa. In addition, the increase in histone deacetylation would lead to histone methylation in specific positions involved in gene repression and silencing, such as H3K9 [54,55,56,57].

In any case, considering that spermatozoa are produced continuously, we have no evidence that the abnormal protein patterns observed in samples isolated from men living in the “Land of Fires” cannot change over time because of the continuous changes in environmental conditions and in the quantity or types of xenobiotics accumulated in gametes.

In conclusion, in this work, we demonstrated for the first time the involvement in DNA oxidative damage of human SNBP from men exposed to pollutants, giving new insights on the toxicity mechanisms of some heavy metals. The potential implications of these findings could provide guidance in the future to better understand many mechanisms related to different diseases and processes in which oxidative DNA damage is implicated.

## 4. Materials and Methods

### 4.1. Reagents

All used reagents were of analytical grade and purchased at Sigma-Aldrich (Merck KGaA, Darmstadt, Germany).

### 4.2. Ethical Statements

All methods were carried out in accordance with the Code of Ethics of the World Medical Association (Declaration of Helsinki) guidelines and regulations. All experimental protocols were approved by the Ethical Committee of the Local Health Authority Campania Sud-Salerno (Committee code 43/2015/06). Informed consent was obtained from all recruited subjects (over 18) before sample collection.

### 4.3. Recruitment

The recruitment was conducted from October 2017 to November 2018, during a pilot study (EcoFoodFertility initiative, www.ecofoodfertility.it) to investigate the use of human semen as an early biomarker of pollution in healthy men [11,12,58], living in areas with low and high environmental impact in the Campania region (Southern Italy). The geographical areas selected for the recruitment are shown in Figure 1. Semen samples from the first group (*n* = 80 healthy males) came from San Francesco d’Assisi Hospital in Oliveto Citra-Province of Salerno, which is a municipality belonging to the low environmental impact area known as “Alto-medio Sele” (Oliveto Citra, Contursi Terme, San Gregorio Magno, Buccino, Ricigliano, Valva, and Colliano). This area has a low environmental impact (https://www.arpacampania.it/); its economy is principally based on low-to-medium scale farming and without known illegal disposal of toxic wastes (green circle in Figure 1). The semen samples of the second group (*n* = 160 healthy males) came from the municipalities belonging to the “Land of Fires” (Acerra, Caivano, Afragola, Casalnuovo, Pomigliano d’Arco, Brusciano, Giugliano, Cardito, and Marigliano) and the Medicina Futura center (Acerra-Province of Naples) (red circle in Figure 1). “Land of Fires” is a high environmental impact area of Campania that is officially recognized on the basis of the Campania Region Environmental Protection Agency report, as an area with the highest concentration of illegal disposal sites of toxic waste (https://www.arpacampania.it/). The participants were selected within clinically healthy male volunteers in fertile age (in the range 18–30 years old). Enrollment criteria were as follows: residence for at least 10 years in the study area, no known chronic diseases (diabetes or other systemic diseases), no varicocele, no prostatitis, and other factors that could affect semen quality (such as fever, medications, exposure to X-rays, etc.), no drinker, no smoker, no reported history of drug abuse, and no known occupational exposures to toxic chemicals. Moreover, the recruited participants follow a healthy diet and practice at least 30 min of walking at day. Data were collected by questionnaire and physical examination, including the urogenital evaluation (testis volume and transrectal prostate evaluation). Upon enrolment, a code number (HS1, HS2, HS3, …, HSn) was assigned to each volunteer by the recruiting andrologist (the recruiter) in order to preserve anonymity. Each code number was uploaded into a computer database along with personal and clinical information.

### 4.4. Spermatozoa Collection and SNBP Extraction

Semen samples were centrifuged at 5500 × *g* for 30 min at 4 °C in order to separate the spermatozoa from seminal plasma. Sperm pellets with a volume of about 50 µL were stored at −80 °C until biochemical and molecular analyses. The protocol used for SNBP extraction is based on the reports from [59], with slight changes. In brief, the sperm pellets were washed twice with 500 µL of phenylmethanesulfonyl fluoride (PMSF), resuspended with 50 µL of 1 mM PMSF and 50 µL of a solution containing 6 M guanidinium chloride and 10 mM DTT and then incubated at 20 °C for 30 min. For sperm chromatin precipitation, 5 volumes of cold absolute ethanol were added, and the samples were incubated at least 60 min at –20 °C. After centrifugation at 13,680 g for 15 min at 4 °C, the pellet obtained was resuspended in 500 µL of 0.5 M of HCl; the sample was incubated for 5 min at 37 °C and then centrifuged a 1000× *g* for 10 min at 4 °C. The sperm nuclear basic proteins were precipitated from supernatant obtained by adding trichloro acetic acid with a final concentration of 20%; the sample was incubated 60 min at 4 °C, and then centrifuged at 14,000× *g* for 10 min at 4 °C. The pellet obtained was washed by adding 500 μL of acetone containing 1% β-mercaptoethanol. The sample was centrifuged twice at 14,000× *g* for 10 min at 4 °C and the final pellet dried in a speed-vacuum for 10–15 min or under a fume hood at room temperature. The dried proteins were resuspended in 50 μL of ultrapure water (milliQ) and used immediately or stored at −20 °C in aliquots of 50 µg.

### 4.5. Acid-Urea Polyacrylamide Gel Electrophoresis

Human protamines were analyzed by AU-PAGE as previously described [17]. In brief, the components of gel were 15 mL of a solution composed by 2.5 M urea, 0.9 M acetic acid, and 15% acrylamide/0.1% N,N’-Methylene-bis-acrylamide, 80 µL of TEMED and 800 µL of 10% APS. After gelification in about 1 h, at room temperature, pre-electrophoresis was performed at 150 V for 1 h 30 min, placing the negative electrode at the bottom of the gel. The buffer used was 0.9 N acetic acid and in the wells were loaded 20 µL of a solution containing 0.9 N acetic acid 2.5 molar urea. After pre-electrophoresis, the wells were washed with 0.9 N acetic acid buffer using a syringe, and the electrophoretic chamber was filled with fresh 0.9 N acetic acid buffer. Then, 2–2.5 µL per well of each sample, containing 4 µg of proteins in 0.9 N acetic acid and 2.5 molar urea, were loaded for the run, which was conducted at 150 V for about 55 min. At the end of the electrophoresis, gels were stained with Amido Black, and then with Coumassie Blue Brilliant R-250, as previously described [60]. Gels were acquired using a Gel-Doc system (BioRad, Hercules, CA, USA) through Quantity One v.4.4.0 (BioRad, Hercules, CA, USA) software. A densitometric analysis of the bands on the gel was performed using the software ImageJ ver 1.50d (Wayne Rasband, National Institute of Health, Bethesda, ML, USA, https://imagej.nih.gov/ij/, 1997–2018).

### 4.6. Plasmid DNA Preparation

pGEM3 plasmid DNA (2867 bp) was purified from transformed *Escherichia coli* HB 101 cells, using the method as previously described [61]. For plasmid purification, we used the standard protocol of the QIAGEN Plasmid Midi Purification kit (QIAGEN Plasmid Midi Purification handbook, third edition © 2005) but performing all the steps at 12 °C and not at 22 °C as recommended by Qiagen handbook in order to obtain high amounts of sc pDNA. Another critical step was the pDNA pellet air drying after isopropanol precipitation and ethanol washes. During these steps, we maintain the sample on ice avoiding pipetting DNA, because this may cause shearing. Finally, the quality of plasmid DNA was evaluated by gel electrophoresis on 1% agarose gels in 89 mM Tris-HCl pH 8.0, 2 mM EDTA, and 89 mM boric acid (TBE). The obtained plasmid DNA was used, in the circular form, for Electrophoretic Mobility Shift Assays (EMSA) of DNA and DNA oxidative damage experiments.

### 4.7. DNA Binding Affinity of SNBP by EMSA

The effect of human SNBP, extracted from individuals living in low and high environmental impact area, on DNA was analyzed by EMSA as previously described [62], with slight modifications. In brief, mixtures DNA/proteins were prepared. Each mixture contained 150 ng of plasmid DNA (pGEM3) and an increasing amounts of proteins, which was expressed as protein/DNA wt/wt ratios (reported on the wells of the gels shown in the results section). The protein/DNA wt/wt ratios were between 0.05 and 3.8, as indicated in each experiment. At the end of the interaction between DNA and proteins (5 min at room temperature), all samples were added with TBE 10× (to obtain TBE 1× final concentration) just before running the gels and analyzed on 1% agarose gel in TBE. DNA migration was visualized by staining agarose gels with ethidium bromide (2 µg/mL) after electrophoresis. All experiments were performed at least five times.

### 4.8. Fluorescence Spectroscopy

The fluorescence analyses were carried out in a 1 cm optical path length cuvette (STARNA), 0.5 mL volume, using a Jasco spectrofluorometer model FP 8200, equipped with a Julabo F25-HD temperature controller (Julabo GmbH, Seelbach, Germany). Fluorescence measurements has been carried out on human protamines at the concentrations of 0.025 mg/mL in the presence of 5 µM ANS in water. Fluorescence spectra were acquired in the emission range of wavelength from 420 to 600 nm after excitation at 350 nm. Photomultiplier absorbance did not exceed 600 V in the spectral regions measured. Each spectrum was signal averaged at least three times and smoothed with the software Spectra Manager Ver. 2.09 (Jasco Analytical Instruments, Tokyo, Japan). All measurements were performed at least three times at 25 °C.

### 4.9. DNA Breakage Analyses

pGEM3 plasmid DNA breakage in the presence of SNBP extracted from individuals living in low and high environmental impact area and 30 μM hydrogen peroxide (H_2_O_2_) was analyzed on 1% agarose gel in TBE 1× final concentration. Here, 150 ng of plasmid DNA (pGEM3) and proteins/DNA *w*/*w* ratios in a range from 0.1 to 0.4 were used. DNA and proteins were incubated at room temperature for 5 min in order to interact, after which H_2_O_2_ was added and the samples were incubated for 30 min at 37 °C in the dark. At the end of incubation, samples were added with TBE in order to obtain 1× final concentration just before electrophoresis analysis in order to avoid the EDTA coordination of eventual metals. Electrophoresis was carried out at 100 V for 30 min. DNA migration was visualized by staining agarose gels with ethidium bromide (2 μg/mL) after electrophoresis. All experiments were performed at least five times.

### 4.10. Aniline Blue Staining

The staining was performed as previously described [63], with few modifications. In brief, the fresh semen smear of each sample was air dried and then stained with 5% aqueous aniline blue stain (Histon Color Test, AB Analitica, Padua, Italy) in 4% acetic acid (pH 3.5) for 5 min. A cover slide 24 × 50mm was put on each slide. Stained and unstained spermatozoa were observed using light microscopy (Nikon Eclipse Ci) at ×1000 magnification under oil immersion (Plan 100×/1.25 oil objective).

### 4.11. Densitometric Gel Analysis

Gels were acquired using a Gel-Doc system (BioRad, Hercules, CA, USA) via Quantity One v.4.4.0 (BioRad, Hercules, CA, USA) software. Densitometric analysis on gel bands was carried out using the software ImageJ ver 1.50d (https://imagej.nih.gov/ij/) supported by the National Institute of Health (Wayne Rasband, National Institute of Mental Health). The quantification of DNA binding saturation by SNBP measured by EMSA was calculated by subtracting the value obtained from the densitometric analysis of each supercoiled bands to the value in the absence of proteins. The quantification of DNA oxidative damage from bands on agarose gel was determined as the percentage of the band of relaxed DNA form with respect to the total amount of DNA (supercoiled + relaxed forms) bands.

## Figures and Tables

**Table 1:** List of municipalities in low and high environmental impact areas for the recruitment.

Municipalities	
Low Impact Areas	High Impact Areas
Oliveto Citra–Contursi Terme–San Gregorio Magno–Buccino–Ricigliano–Valva–Colliano	Acerra–Caivano–Afragola–Casalnuovo-Pomigliano d’Arco–Brusciano–Giugliano–Cardito–Marigliano

## Supplementary Materials

Supplementary materials can be found at https://www.mdpi.com/1422-0067/21/12/4198/s1, Figure S1: DNA binding ability of sperm proteins obtained from L-group analyzed by EMSA; Figure S2: Evaluation of pGEM3 DNA plasmid breakage in presence of H_2_O_2_ concentrations; Figure S3: DNA breakage induced by H_2_O_2_; Figure S4: DNA binding ability of SNBP in the presence of 15 µM CuCl_2_ analyzed by EMSA; Figure S5: DNA breakage induced by H_2_O_2_ in the presence of 15 µM CuCl_2_; Figure S6: Graphical representation of oxidative DNA damage in presence of human sperm proteins.

## Abbreviations

ROS	Reactive oxygen species
AU-PAGE	Acid-urea Polyacrylamide Gel Electrophoresis
EMSA	Electrophoretic Mobility Shift Assays
ANS	8-Anilinonaphthalene-1-sulfonic acid
SNBP	Sperm Nuclear Basic Proteins
H-group	Man living in the high environmental impact areas
L-group	Man living in the low environmental impact areas
CP/Hr	Canonical protamines/histones ratio
nCP/Hr	Not canonical protamines/histones ratio
only-H	Only histones and other basic proteins
DFI	DNA fragmentation index
