# Supplementary material for: Discovery of the Involvement in DNA Oxidative Damage of Human Sperm Nuclear Basic Proteins of Healthy Young Men Living in Polluted Areas
Source: Int J Mol Sci. 2020 Jun 12;21(12):4198. doi: 10.3390/ijms21124198 (PMC7349829; doi:10.3390/ijms21124198)
Supplement: Supplementary file 1 [file ijms-21-04198-s001.pdf]

Supplementary materials

Supplementary Figures

Figure S1. DNA binding ability of sperm proteins obtained from L-group analyzed by EMSA.

Figure S2. Evaluation of pGEM3 DNA plasmid breakage in presence of H_2O_2 concentrations.

Figure S3. DNA breakage induced by H_2O_2.

Figure S4. DNA binding ability of SNBP in the presence of 15 μM $CuCl_2$ analyzed by EMSA.

Figure S5. DNA breakage induced by H_2O_2 in the presence of 15 μM $CuCl_2$.

Figure S6. Graphical representation of oxidative DNA damage in presence of human sperm proteins.

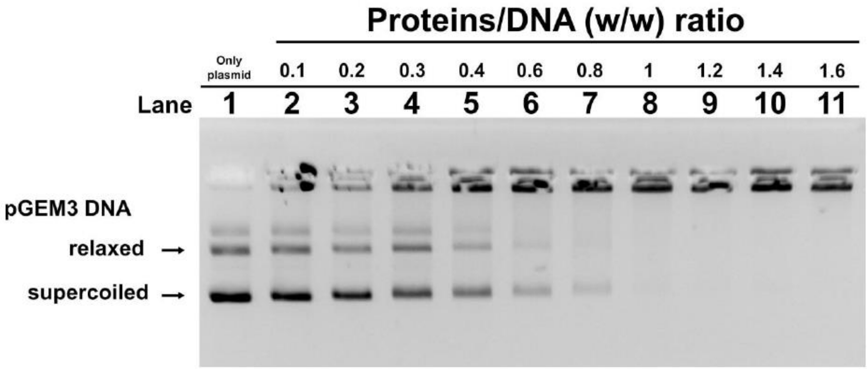

Figure S1. DNA binding ability of sperm proteins obtained from L-group analyzed by EMSA on 1% agarose gel. Bands on gel representing the state of pGEM3 plasmid DNA incubated in a w/w ratio with increasing amount (0.1 to 1.6) of sperm proteins from samples containing CP/Hr.

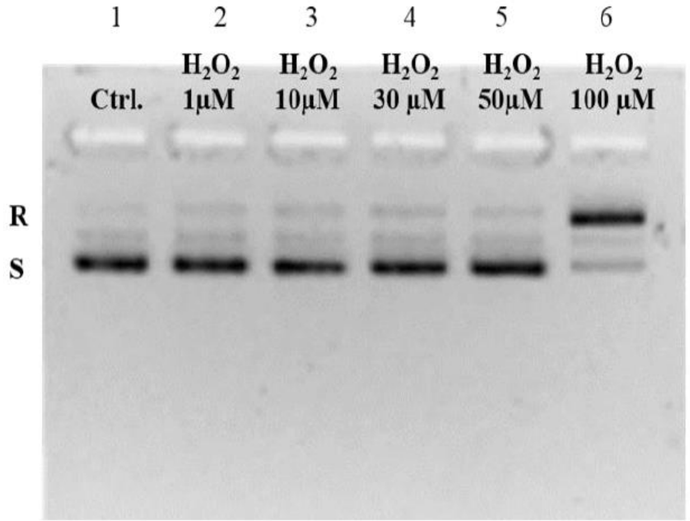

Figure S2. Evaluation of pGEM3 DNA plasmid breakage in presence of H_2O_2 concentrations. DNA breakage is evaluated by the conversion of supercoiled (S) to relaxed (R) form of 150 ng of circular pGEM3 DNA plasmid in absence (Ctrl.) and in presence of increasing concentration of H_2O_2 (from 1 to 100 μM).M).

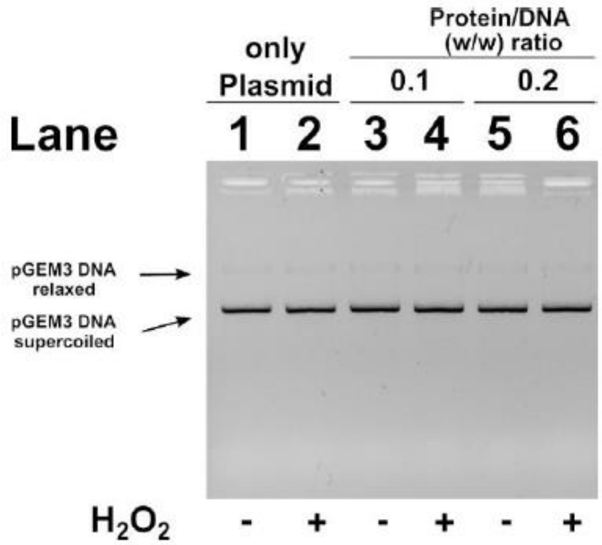

Figure S3. Analysis on 1% agarose gel of pGEM3 plamid DNA breakage induced by H_2O_2, in the presence of SNBP from L-group samples showing the CP/Hr.

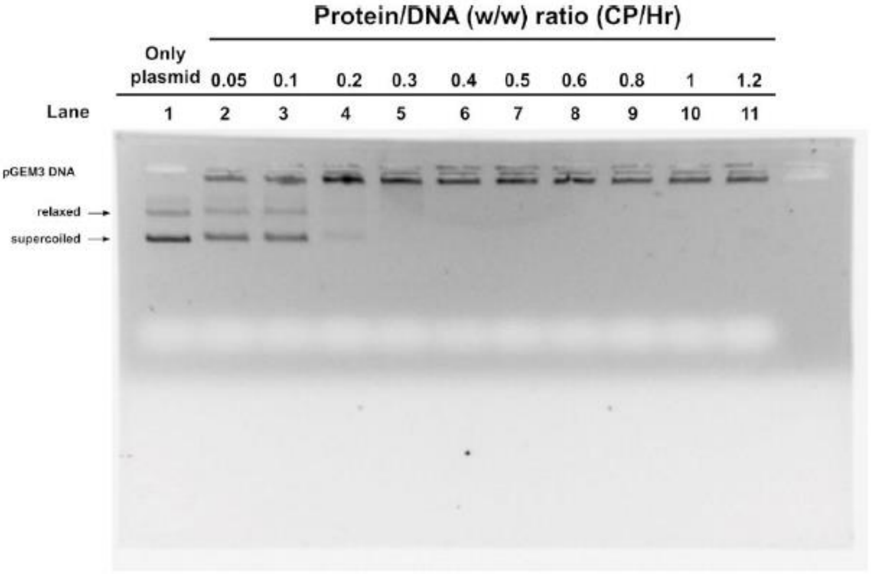

Figure S4. DNA binding ability of SNBP in the presence of 15 μM $CuCl_2$ analyzed by EMSA on 1% agarose gel. Bands on gel representing the state of pGEM3 plasmid DNA incubated in a ratio w/w with increasing amount of SNBP from L-group and containing CP/Hr.

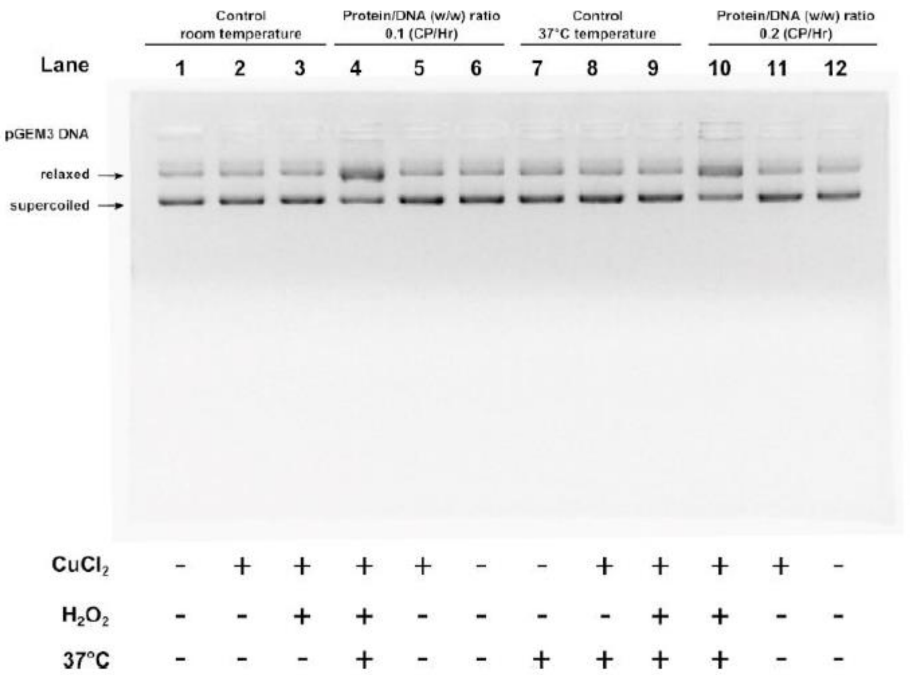

Figure S5. Analysis on 1% agarose gel of pGEM3 plamid DNA breakage induced by H_2O_2. In the presence of SNBP from L-group samples showing CP/Hr after addition of $CuCl_2$.

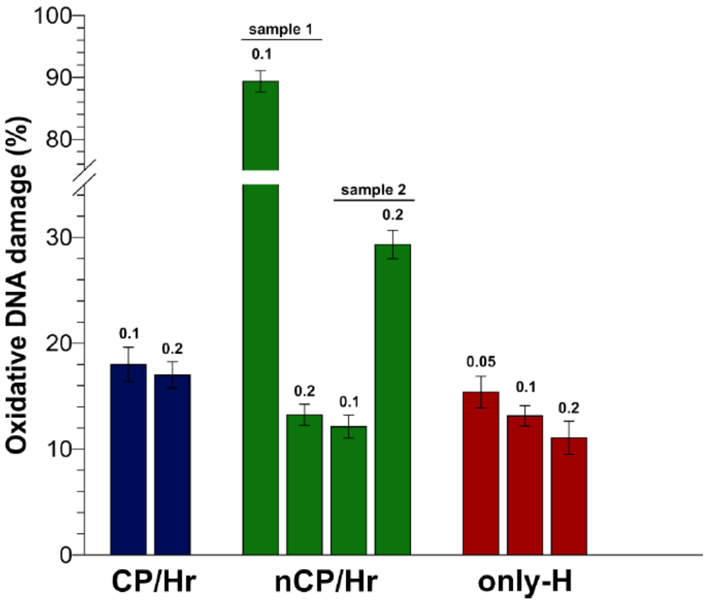

Figure S6. Graphical representation of oxidative DNA damage in presence of human sperm proteins from samples belonging to H-group showing CP/Hr, nCP/Hr, and only-H at different protein/DNA *w/w* ratio quantified by densitometric analysis of bands on gels in Figure 5.
